# Efficacy and Safety of Procalcitonin-Guided Antibiotic Therapy in Lower Respiratory Tract Infections

**DOI:** 10.3390/antibiotics2010001

**Published:** 2013-01-22

**Authors:** Daniel Drozdov, Frank Dusemund, Beat Müller, Werner C. Albrich

**Affiliations:** Medical University Department, University of Basel, Kantonsspital Aarau, Tellstrasse, CH-5001 Aarau, Switzerland; E-Mails: Frank.Dusemund@kssg.ch (F.D.); Beat.Mueller@ksa.ch (B.M.); Werner.Albrich@ksa.ch (W.C.A.)

**Keywords:** isrctn.org **Identifier**: ISRCTN40854211, procalcitonin, biomarkers, lower respiratory tract infections, antibiotic stewardship

## Abstract

**Background:** In 14 randomized controlled studies to date, a procalcitonin (PCT)-based algorithm has been proven to markedly reduce the use of antibiotics along with an unimpaired high safety and low complication rates in patients with lower respiratory tract infections (LRTIs). However, compliance with the algorithm and safety out of controlled study conditions has not yet been sufficiently investigated. **Methods:** We performed a prospective international multicenter observational post-study surveillance of consecutive adults with community-acquired LRTI in 14 centers (Switzerland (n = 10), France (n = 3) and the United States (n = 1)). **Results:** Between September 2009 and November 2010, 1,759 patients were enrolled (median age 71; female sex 44.4%). 1,520 (86.4%) patients had a final diagnosis of LRTI (community-acquired pneumonia (CAP), 53.7%; acute exacerbation of chronic obstructive pulmonary disease (AECOPD), 17.1%; and acute bronchitis, 14.4%). Compliance with the PCT-guided therapy (overall 68.2%) was highest in patients with bronchitis (81.0% *vs*. AECOPD, 70.1%; CAP, 63.7%; *p* < 0.001), outpatients (86.1% *vs*. inpatients, 65.9%; *p* < 0.001) and algorithm-experienced centers (82.5% *vs*. algorithm-naive, 60.1%; *p* < 0.001) and showed significant geographical differences. The initial decision about the antibiotic therapy was based on PCT value in 72.4%. In another 8.6% of patients, antibiotics were administered despite low PCT values but according to predefined criteria. Thus, the algorithm was followed in 81.0% of patients. In a multivariable Cox hazard ratio model, longer antibiotic therapy duration was associated with algorithm-non-compliance, country, hospitalization, CAP *vs*. bronchitis, renal failure and algorithm-naïvety of the study center. In a multivariable logistic regression complications (death, empyema, ICU treatment, mechanical ventilation, relapse, and antibiotic-associated side effects) were significantly associated with increasing CURB65-Score, CAP *vs*. bronchitis, multilobar pneumonia, but not with algorithm-compliance. **Discussion:** Cultural and geographic differences in antibiotic prescribing affected the compliance with our PCT-guided algorithm. Efforts to reinforce compliance are needed. Antibiotic stewardship with PCT is possible, effective and safe without increasing the risk of complications in real-life conditions.

## 1. Introduction

The efficacy and safety of procalcitonin (PCT)-guided antibiotic stewardship in lower respiratory tract infections (LRTIs) has been documented in several randomized controlled trials (RCTs) [1,2,3,4,5,6,7]. In these studies the start or discontinuation of antibiotics was recommended or discouraged based on PCT cut-off values. The total antibiotic exposure was reduced by 25%–75% depending on diagnosis and site of care without an increase in morbidity or mortality. The greatest reduction was achieved by fewer initiations of antibiotic therapy in viral bronchitis in ambulatory patients. In sicker hospitalized patients with CAP, the most marked reduction was achieved mainly by early discontinuation and thus shortening of antibiotic courses.

Results from RCTs may not unconditionally be extended to daily use because of the possibility of unexpected negative effects in the implementation of the study concept, selective in- and exclusion of study patients, limited validity or the lack of practicability, respectively. Except for a single center post study surveillance at the cantonal hospital of Aarau, Switzerland [8] and a study in an ambulatory setting in Germany [7], there are no data about the real life efficacy of PCT-guided antibiotic stewardship for LRTI. 

In this context, we performed a prospective, observational, multi-center, international quality control survey to assess the PCT-guided antibiotic stewardship in in-and outpatients with LRTIs and to show that the results can be generalized [9]. In this paper we provide insights in this study with the focus on safety and compliance with the prespecified algorithm. 

## 2. Methods

Between September 2009 and February 2011 we enrolled consecutive patients with LRTI presenting to emergency rooms or practitioner’s offices in centers in Switzerland (n = 10), France (n = 3) and the United States (n = 1) in a prospective, observational, international, multi-center, quality control survey. Three of the Swiss hospitals had previous experience with the PCT algorithm (“algorithm experienced centers”), all others were considered algorithm naive (“PCT naive centers”). LRTIs (acute bronchitis, acute exacerbation of chronic obstructive pulmonary disease (AECOPD), community acquired pneumonia (CAP) as well as the severity of the COPD were defined according to guidelines [10].

Diagnostic workup and treatment were left to the responsibility of the treating physicians. Measurement of PCT levels was recommended in all patients on admission and for inpatients every 2 to 3 days as long as they received antibiotic treatment. PCT measurement was available daily around the clock using highly sensitive immunoassays according to the different centers availability (Kryptor®, BRAHMS AG, Hennigsdorf, Germany or Vidas®, BioMérieux, Marcy l'Etoile, France). These results were available in approximately one hour. 

**Figure 1:**
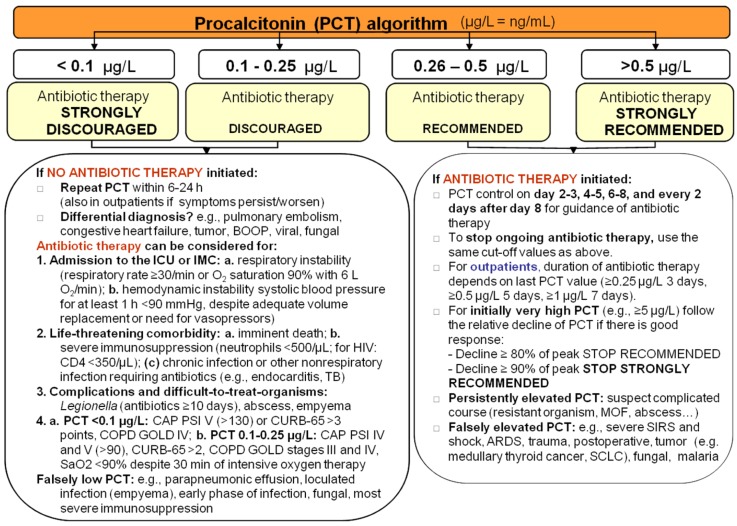
Algorithm for procalcitonin (PCT)-guided antibiotic therapy. ARDS, acute respiratory distress syndrome; BOOP, bronchiolitis obliterans with organizing pneumonia; CAP, community-acquired pneumonia; COPD GOLD, chronic obstructive pulmonary disease Global Initiative for Chronic Obstructive Lung Disease; CURB-65, confusion, serum urea nitrogen, respiratory rate, blood pressure, and age 65 years or older; HIV, human immunodeficiency virus; ICU, intensive care unit; IMC, intermediate care unit; MOF, multiple organ failure; PSI, Pneumonia Severity Index; SCLC, small-cell lung cancer; SIRS, systemic inflammatory response syndrome; and TB, tuberculosis.

All patients were registered by the physician on duty (or in the US center by study nurses) on a password-secured website. The website displayed the recommended and previously published PCT algorithm and the cut-off values (Figure 1) [9]. 

Study personnel (in the USA) and physicians were instructed in initial face-to-face 1-hour seminars about the quality control survey, the website and the treatment algorithm. Throughout the study, weekly reminder e-mails were sent to study personnel and physicians.

According to the predefined overruling criteria antibiotic therapy was permitted by the algorithm despite of low PCT levels but not compulsory. These included the following: admission to the intensive care unit, life-threatening comorbidity, complications of LRTI (abscess or empyema), difficult-to-treat organisms (e.g., Legionella) or high clinical severity scores (Pneumonia Severity Index (PSI), CURB65) (Figure 1). 

The primary end point of the study was the total duration of antibiotic treatment within 30 days [9]. In this analysis we investigated the reasons for non-adherence to the PCT algorithm, the duration of antibiotic therapy during the initial presentation and the risk of complications within 30 days. 

Discrete variables were expressed as counts (percentage) and continuous variables as medians and interquartile ranges (IQR), unless stated otherwise. Frequency comparison was done by the Chi-square test. Two-group comparison of normally distributed data was performed by Student’s t-test. For data not normally distributed, the Mann-Whitney-U-test was used. In order to assess independent risk factors for complications we used a generalized linear model including 16 variables with a binomial distribution and a logit link, which represents a logistic regression model.

The Swiss and French local ethics committees considered this study as a quality control survey; only at the US site was an informed consent necessary from all the patients. 

## 3. Results

1810 patients were registered on the website. Of those, 1759 had complete data sets from the index visit (Switzerland: 1361; USA: 295; and France: 103). Of 1520 patients (86.4%) with a final diagnosis of LRTI, 1425 (93.8%) had sufficient follow-up information at day 30 after enrolment and constitute the main analysis population. 44.4% of patients were female, the median age was 71.0 years. The final diagnosis was in 53.7% CAP, in 17.1% AECOPD and in 14.4% acute bronchitis. 173 (11.4%) patients were enrolled as outpatients. 71.3% had at least one comorbidity [9].

Of 1520 patients with LRTIs, 1208 (79.5%) received antibiotics with a mean duration of antibiotic therapy of 6.9 days (IQR: 2–10 days). Algorithm compliance was higher in algorithm experienced (82.5%) than algorithm-naive centers (60.1%; *p *< 0.0001). It was highest in patients with bronchitis and influenza (81%), followed by AECOPD (70.1%) and CAP (63.7%). There were remarkable differences between countries and treatment sites. The algorithm compliance in outpatients in France (85.1%) and outpatients in Switzerland (87.6%) was similar (*p* = 0.63). In inpatients the compliance showed a great variability with 33.5% in the USA, 66% in France and 74.5% in Switzerland (*p* < 0.001 for USA *vs*. France or Switzerland; *p* = 0.06 for France *vs*. Switzerland) [9]. 

**Figure 2:**
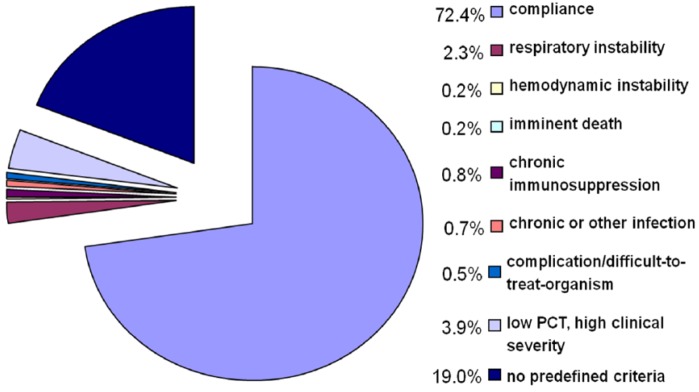
Compliance and overruling reasons.

Antibiotic therapy strictly followed PCT cut-off ranges on initial presentation in 72.4% of patients. In 8.6% of patients predefined overruling criteria were applied (Figure 1), resulting in an overall algorithm compliance of 81.0% (Figure 2). 

The most important overruling reasons were high clinical severity (CURB65, PSI by patients with CAP and GOLD class by patients with AECOPD) in 3.9% and respiratory instability (respiratory rate ≥30/min or O_2_ saturation <90% with 6 L O_2_/min) in 2.3% (Figure 2). In 19.0% of patients antibiotic therapy was prescribed due to clinical judgment only despite of low PCT levels and without prespecified overruling reasons. During the entire index presentation (practitioner’s office visit, emergency room visit or entire hospitalization by inpatients), overall algorithm compliance was 68.2%.

**Figure 3:**
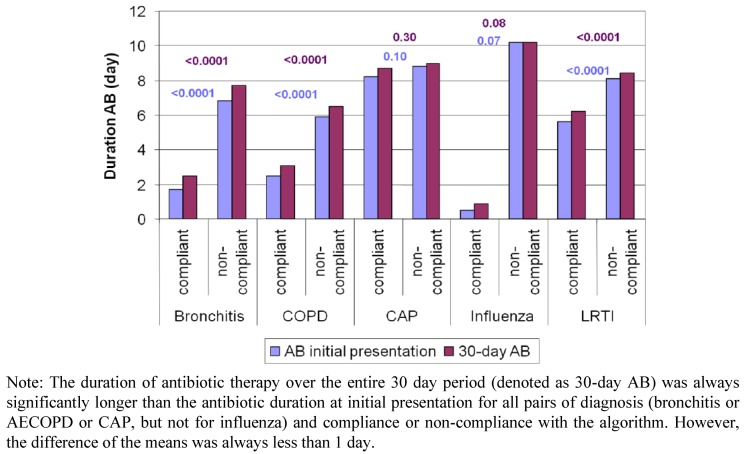
Mean duration of antibiotic therapy, diagnosis and algorithm compliance.

The overall mean duration of antibiotic therapy was 6.9 days (IQR: 2–10) with significant differences between diagnoses (acute bronchitis: 3.5 days, AECOPD: 4.1 days, CAP: 8.8 days; *p* ≤ 0.001 for all comparisons). The duration of antibiotic therapy was significantly shorter in case of algorithm adherence than non-adherence (6.2 days *vs*. 8.4 days; *p* < 0.001). After stratification for diagnosis this trend was found as well in patients with bronchitis and AECOPD, but in patients with CAP and influenza the trend was not significant (Figure 3). Algorithm compliance was an independent significant predictor for a shorter duration of antibiotic therapy in a Cox proportional hazards model (Table 1). In contrast, CAP (*vs*. bronchitis), treatment in France (*vs*. Switzerland), in-hospital (*vs*. ambulatory) treatment, renal insufficiency, as well as treatment in a PCT naive center were associated with longer antibiotic therapy duration. 

**Table 1:** Predictors of antibiotic therapy duration within 30 days.

Cox proportional hazard ratio			
30-day antibiotic duration	HR	95% CI	P
Compliance	1.27	1.13–1.43	<0.0001
CAP (*vs*. bronchitis)	0.53	0.45–0.62	<0.0001
France (*vs*. Switzerland)	0.66	0.55–0.79	<0.0001
Hospital treatment	0.62	0.52–0.75	<0.0001
Algorithm naive	0.86	0.76–0.97	0.015
Renal insufficiency	0.82	0.72–0.93	0.003

HR > 1 denotes shorter, HR < 1 longer antibiotic duration.

In a multivariate logistic regression analysis algorithm compliance was not associated with a higher risk for complications (death, pleural empyema, ICU, mechanic ventilation, relapse, side effects of antibiotics) within 30 days. Significant risk factors for complications were an increasing CURB-65 score, the diagnosis of CAP (*vs*. bronchitis) and multi-lobar pneumonia, while a previous stroke was associated with a lower risk (Table 2). 

**Table 2:** Risk factors for complications within 30 days.

30 day complications	OR	95% CI	P
CURB65	1.35	1.07–1.72	0.01
CAP (*vs*. bronchitis)	6.81	2.97–15.61	<0.0001
Multilobar pneumonia	5.25	1.62–17.02	0.006
History of stroke	0.25	0.08–0.77	0.015

Compliance with the algorithm was not associated with risk of complication (*p* = 0.26).

Since the majority of centers and patients were in Switzerland with only a single US center and a small fraction of outpatients, the generalizability is limited. 

## 4. Conclusions

In this international multi-center post study survey of PCT-guided antibiotic therapy for LRTI, being the largest to date, we show that after short introductory seminars, our recommended algorithm was suitable and had a high compliance-rate for daily use in real-life conditions [9]. This fact was previously only shown in small studies [4,7,8]. Antibiotic therapy can be individualized and notably reduced (bronchitis, AECOPD) or significantly shortened (CAP) [11]. Good algorithm compliance was essential and led to a significantly shorter antibiotic therapy duration. The algorithm compliance was remarkably high with 81% on initial presentation and with 68% during the entire index presentation. In contrast, in the EDCAP study for patients with low PSI scores on admission, outpatient treatment was recommended. However, the compliance with these recommendations was only 37% [12,13]. 

The use of antibiotics [14] and the algorithm compliance were strongly affected by cultural and geographic factors. Not surprisingly the compliance was best in Switzerland, where antibiotic use and antibiotic resistance are considerably lower than in France and in the USA [15]. A clear correlation between the use of antibiotics and antibiotic resistance was confirmed in an earlier study [16]. The algorithm compliance was the greatest in patients with bronchitis and in outpatients, *i.e.*, patients with particularly high probabilities for viral infection. Importantly, algorithm compliance did not lead to higher complication rates, which could serve as an important argument for the safety of our concept. The predefined criteria permitting antibiotic therapy despite low PCT levels, certainly contributed to the safety of our algorithm. 

Limitations of clinical and laboratory tests and especially biomarkers have to be considered. In daily practice it is important to be well aware of the kinetics of PCT as well as situations with false-high and false-low results (Figure 1) [17]. While highly elevated PCT levels were found in patients with pneumococcal CAP [18], the same was not true in CAP due to atypical organisms such as mycoplasma [19], where the impact and necessity of antimicrobial therapy is debated. Furthermore antimicrobial pre-treatment, parapneumonic effusion, loculated infection (empyema), early phase of infection and most severe immunosuppression may lead to lower PCT levels [20]. Unspecific elevations of PCT levels in the absence of a bacterial infection can typically be seen in situations of massive stress, for example in severe trauma, postoperative, severe SIRS, shock, acute respiratory distress syndrome, but also in patients with tumor (e.g. medullary thyroid cancer, small cell lung cancer) or fungal infections and malaria [21,22,23].

However, there is no other biomarker including CRP with such an extensive safety and efficacy record as PCT, notably in patients with LRTI and sepsis [11,24,25]. In a meta-analysis, the superiority of PCT over CRP has already been shown [26]. 
